# Role of primary protectors of plant cells in salinity tolerance: molecular mechanisms and adaptive strategies

**DOI:** 10.1080/15592324.2026.2687962

**Published:** 2026-06-12

**Authors:** Susmita Das, Edappayil Janeeshma, Hesam Mousavi, Henrik Aronsson, Mohammad Sarraf

**Affiliations:** a Department of Microbiology and Biotechnology, Sister Nivedita University (SNU), Newtown, West Bengal, India; b Department of Botany, MES Keveeyam College, Malappuram, Kerala, India; c Faculty of Applied Ecology, Agricultural Science, and Biotechnology, University of Inland Norway, Elverum, Norway; d Department of Biological and Environmental Sciences, University of Gothenburg, Gothenburg, Sweden; e Department of Horticultural Science, Faculty of Agriculture, Shahid Chamran University of Ahvaz, Ahvaz, Iran

**Keywords:** Salinity stress, Na⁺/K⁺ homeostasis, osmolytes, antioxidants, stress proteins

## Abstract

Salinity stress is among the most pervasive abiotic factors limiting plant growth and agricultural productivity worldwide, currently affecting more than 20% of irrigated croplands. Elevated salt concentrations induce osmotic imbalance, ionic toxicity, and oxidative stress, leading to disruptions in photosynthesis, metabolism, and plant development. To counteract these deleterious effects, plants have evolved intricate, highly coordinated defense systems comprising structural and functional cellular protectors. At the structural level, the cell wall, plasma membrane, vacuole, and peroxisome play critical roles in maintaining cellular homeostasis under saline conditions. These cellular structures safeguard the cell by regulating ion fluxes, preserving membrane integrity, and mitigating the toxicity of reactive oxygen species (ROS). Ion transporters such as SOS1, NHX1, and HKT1, along with vacuolar proton pumps, maintain optimal Na⁺/K⁺ balance and pH regulation within cellular compartments. Functionally, plants deploy osmolytes and compatible solutes, including proline, glycine betaine, trehalose, and polyamines that act as osmoprotectants, stabilizing proteins and membranes while maintaining cellular turgor. Concurrently, robust antioxidant systems, comprising both enzymatic components (superoxide dismutase, catalase, ascorbate peroxidase) and non-enzymatic molecules (ascorbate, glutathione, carotenoids), scavenge excess ROS generated during salt stress. Stress proteins such as heat shock proteins (HSPs) and late embryogenesis abundant (LEA) proteins also contribute to proteostasis and membrane stabilization during salt stress. Recent advances (2020–2025) have significantly expanded our understanding of the molecular and signaling networks underlying salinity tolerance. While earlier studies have focused on individual components such as ion transporters, osmolytes, or antioxidant systems, this review uniquely integrates structural organelle-based defenses with functional metabolic responses into a unified framework. We highlight the dynamic cross-communication among transcriptional regulators, ion transport systems, ROS detoxification pathways, and hormonal signaling cascades that collectively orchestrate plant adaptation to salinity. By synthesizing recent findings from a systems-level perspective, this work provides novel insights into the coordinated cellular defense network, identifying key regulatory hubs and multi-target strategies for breeding and biotechnological interventions to improve crop resilience and productivity in salt-affected agroecosystems.

## Graphical abstract

**Figure:**
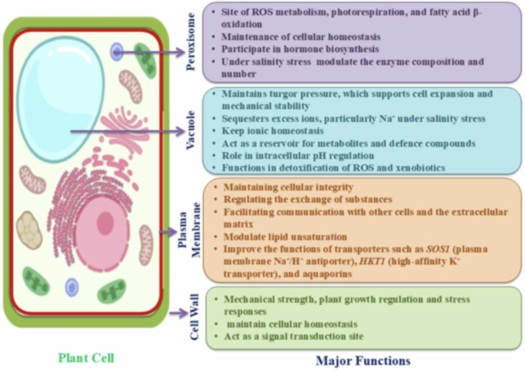

An overview of the major roles of primary protectors in cellular functions. Cellular homeostasis under saline conditions is maintained through the regulation of ion transport, preservation of membrane integrity, and reduction of reactive oxygen species (ROS). These vital functions are carried out by the coordinated action of cellular structures of the cell wall, plasma membrane, vacuole, and peroxisome, which together protect the cell from salt-induced stress.

## 1. Introduction

Soil salinity is an escalating environmental challenge that severely restricts plant productivity and threatens global food security. According to the Food and Agriculture Organization,^1^ more than 1300 million hectares of land are affected by salinity, resulting in significant yield reductions in major crops such as rice, wheat, and maize^1‐3^. Salinity exerts its detrimental effects primarily through osmotic stress, which restricts water uptake, and ionic stress caused by excessive accumulation of Na^+^ and Cl^-^ ions that disrupt cellular metabolism, nutrient balance, and photosynthetic efficiency.^4^ ^,^ ^5^ In addition, salinity-induced oxidative stress enhances the production of reactive oxygen species (ROS), including superoxide radicals (O₂⁻), hydrogen peroxide (H₂O₂), and hydroxyl radicals (•OH), resulting in lipid peroxidation, protein oxidation, membrane damage, and nucleic acid instability.^6^ ^,^ ^7^ These combined effects may cause yield losses of up to 50–70% in salt-sensitive crops.^8‐10^ It triggers chlorosis, reduced leaf area, and impaired root development due to ionic imbalance and water deficit. Salinity adversely affects plants throughout their life cycle, including seed germination, seedling establishment, vegetative growth, flowering, and grain filling. Reproductive stages are often particularly sensitive, resulting in reduced pollen viability, poor fertilization, and diminished seed or grain yield under prolonged salt exposure.^8^

Prolonged exposure leads to membrane destabilization, enzyme inactivation, and metabolic dysfunction.^4^ ^,^ ^11^ At the cellular level, survival under salt stress depends on the ability to maintain cytosolic ion homeostasis, turgor pressure, and redox balance, functions largely mediated by a set of specialized subcellular structures, i.e., primary protectors of the cell.^11‐13^ These structures function as the cellular barriers and response centers that perceive, buffer, compartmentalize, and detoxify salinity-induced damage. Their coordinated activities determine the efficiency of stress perception, ion transport regulation, ROS scavenging, osmotic adjustment, and intracellular signaling during salt stress adaptation. The cell wall, plasma membrane, vacuole, and peroxisome constitute major early-responsive components of the cellular defense network in plants against salinity-induced damage.^6^ ^,^ ^14^ The cell wall acts as a mechanical buffer, adjusting composition (e.g., pectin cross-linking, lignification) to regulate porosity and ion binding.^15^ ^,^ ^16^ The plasma membrane functions as the critical interface for ion sensing, transport, and signaling, involving key components such as SOS3 (Ca²⁺ sensor) for ion sensing, SOS2 (protein kinase) for downstream signaling, and SOS1 (Na⁺/H⁺ antiporter) for Na⁺ extrusion, while also undergoing lipid remodeling to maintain fluidity and integrity.^17^ ^,^ ^18^ The vacuole serves as the major compartment for Na⁺ sequestration via tonoplast-localized Na⁺/H⁺ exchangers (NHX) and proton pumps (V-ATPases), thereby preventing cytosolic toxicity.^19^ ^,^ ^20^ The peroxisome, a central organelle in ROS metabolism, houses key antioxidant enzymes, including catalase (CAT), superoxide dismutase (SOD), and ascorbate peroxidase (APX), which detoxify ROS and maintain cellular redox homeostasis.^21^ ^,^ ^22^

Soil salinization is further intensified by climate change-associated factors such as rising temperatures, irregular precipitation patterns, sea-level rise, and increased evapotranspiration, which accelerate salt accumulation in agricultural soils. In addition, unsustainable irrigation practices, poor drainage systems, and the use of saline groundwater contribute significantly to secondary salinization in cultivated lands, particularly in arid and semi-arid regions.^2^ ^,^ ^10^ These factors are expected to expand salt-affected areas substantially in the coming decades, thereby posing a major challenge to sustainable agriculture and food security.

Although numerous studies and reviews have examined individual aspects of salinity tolerance, including ion transport, ROS detoxification, osmotic adjustment, and organelle-specific stress responses, comparatively little attention has been paid to the coordinated and integrated roles of primary cellular protectors, such as the cell wall, plasma membrane, vacuole, and peroxisome. In particular, the dynamic interactions, signaling coordination, and functional cross-talk among these components under salinity stress remain incompletely understood.

The present review provides an integrated framework for explaining how these primary cellular protectors collectively contribute to salinity adaptation through the coordinated regulation of ion homeostasis, osmotic balance, ROS detoxification, signaling pathways, and cellular protection mechanisms. In comparison with previously published studies, we explored functional crosstalk among the cell's primary protectors. Thus, the evidence reviewed here suggests that plant salinity tolerance depends not on isolated cellular responses but on coordinated interactions among multiple primary cellular protectors. Understanding these integrated defense networks may facilitate the development of crops with improved salinity resilience. However, several proposed signaling and cross-talk mechanisms remain incompletely characterized and require further experimental validation.

## 2. Overview of the primary cellular protectors

The cell wall, plasma membrane, vacuole, and peroxisome collectively function as the primary cellular protectors that maintain plant cellular homeostasis under both normal and stress conditions. In this section, a concise overview of their basic composition and cellular functions is provided. Detailed salinity-induced structural, biochemical, and functional modifications are discussed in Section 4.

### 2.1. Cell wall

The plant cell wall is a dynamic extracellular structure surrounding the plasma membrane that provides mechanical support, maintains cell shape, and mediates environmental interactions.^23^ It serves as the first protective barrier against biotic and abiotic stresses, including pathogen attack, salinity, and drought.^24‐26^ Its structural and functional properties arise from a complex matrix of polysaccharides, structural proteins, and, in specialized cells, lignin. The major polysaccharides include cellulose, hemicellulose, and pectins. Cellulose microfibrils provide tensile strength, while hemicelluloses such as xyloglucans cross-link cellulose fibers to maintain flexibility and cohesion.^23^ Pectins, enriched with galacturonic acid, form a hydrated matrix that supports cell adhesion, porosity, and ion exchange, particularly within the middle lamella.^27^

The cell wall consists of three major layers: the primary wall, secondary wall, and middle lamella. The primary wall is thin and extensible, supporting cell growth, whereas the secondary wall is thicker and lignified, providing mechanical strength and facilitating water transport in specialized tissues. The middle lamella, rich in pectins, binds adjacent cells and maintains tissue integrity. Additionally, stress-induced deposition of compounds such as suberin and callose further strengthens the cell wall and regulates adaptive responses to environmental cues.^24^ ^,^ ^28^ ^,^ ^29^ Beyond providing mechanical support and regulating cell expansion, the cell wall contributes to cell adhesion, intercellular communication, and environmental sensing. Its polysaccharide-rich matrix serves as an interface between the extracellular environment and intracellular signaling systems. Stress-specific remodeling and signaling responses of the cell wall under salinity conditions are discussed in detail in Section 4.1.

### 2.2. Plasma membrane

The plasma membrane is a dynamic and highly specialized structure that separates the cytoplasm from the external environment, maintaining cellular integrity, regulating molecular exchange, and facilitating cell signaling and communication.^30‐32^ It is primarily composed of a phospholipid bilayer, where hydrophilic phosphate heads face the aqueous environments, and hydrophobic fatty acid tails form a semipermeable internal barrier.

Embedded within the bilayer are integral and peripheral proteins involved in transport, signal transduction, enzymatic activity, and cytoskeletal interactions.^33^ Cholesterol molecules regulate membrane fluidity and mechanical stability, ensuring flexibility under changing physiological conditions.^34^ ^,^ ^35^ In addition, membrane-associated glycoproteins and glycolipids contribute to cell recognition, adhesion, and immune-related functions through the glycocalyx layer.

According to the fluid-mosaic model, lipids and proteins exhibit lateral mobility, allowing the membrane to dynamically respond to intracellular and environmental stimuli. Functionally, the plasma membrane controls selective transport of ions, nutrients, and metabolites, mediates signal perception and transduction, and protects cells from mechanical, chemical, and pathogenic stresses.^36^ ^,^ ^37^ Thus, its structural complexity and functional versatility make the plasma membrane a crucial protector of cellular homeostasis and viability.

### 2.3. Vacuole

The plant vacuole is a dynamic and multifunctional organelle that occupies a substantial portion of the mature plant cell, often comprising 80–90% of the cell volume.^38^ ^,^ ^39^ It is enclosed by a single membrane, the tonoplast, which serves as a selective barrier that regulates the exchange of ions, metabolites, and signaling molecules between the cytoplasm and the vacuolar lumen. The vacuolar interior contains a heterogeneous mixture of water, ions (K⁺, Na⁺, Ca²⁺, Cl⁻), organic acids, sugars, amino acids, secondary metabolites, and proteins, which collectively contribute to osmotic balance, storage, and cellular detoxification.^20^

The vacuole plays a central role in plant growth, development, and stress responses. It maintains turgor pressure, which supports cell expansion and mechanical stability, and sequesters excess ions, particularly Na⁺, under salinity stress, thereby contributing to ionic homeostasis. It also serves as a reservoir for metabolites and defense compounds, participates in intracellular pH regulation, and functions in detoxification of ROS and xenobiotics.^40‐42^

### 2.4. Peroxisome

The peroxisome is a small, single-membrane-bound organelle enriched with oxidative enzymes responsible for ROS metabolism, photorespiration, and fatty acid *β*-oxidation. It also plays a crucial role in cellular metabolism and stress responses. They are spherical to oval in shape and contain a dense matrix of enzymes specialized in oxidative reactions, including CAT, SOD, glycolate oxidase, and acyl-CoA oxidases^43^; Peláez.^44^ The peroxisomal membrane regulates the selective import and export of proteins, metabolites, and signaling molecules, ensuring compartmentalization of reactive processes and maintenance of cellular homeostasis.^45^ ^,^ ^46^

Functionally, peroxisomes are central to ROS metabolism, lipid *β*-oxidation, photorespiration, and stress adaptation.^22^ ^,^ ^45^ ^,^ ^47^ They scavenge ROS generated during metabolic activities and environmental stresses, thereby protecting other cellular components from oxidative damage.^48^ ^,^ ^49^ In photosynthetic tissues, peroxisomes collaborate with chloroplasts and mitochondria to mediate photorespiration, converting glycolate into glycine while releasing H₂O₂, which is subsequently decomposed by CAT.^22^ ^,^ ^50^ Peroxisomes also participate in hormone biosynthesis, including the production of jasmonic acid (JA) and indole-3-acetic acid precursors, linking them to plant growth regulation and stress signaling pathways.^51‐54^

## 3. Impacts of salinity stress

**Figure 1.**
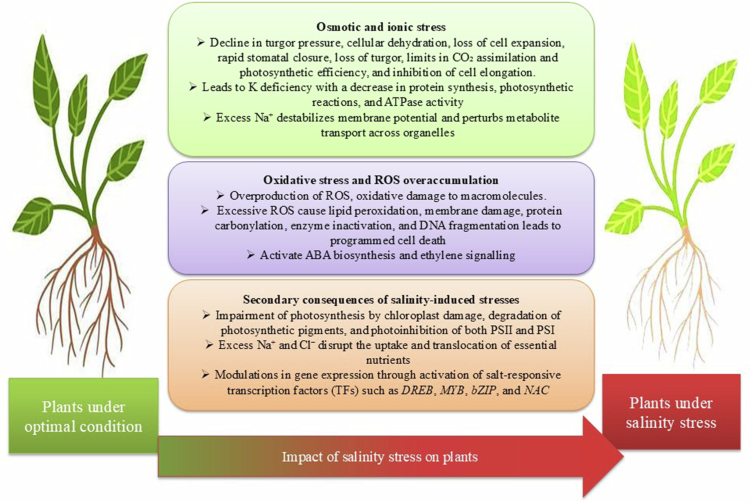
The impacts and secondary consequences of salinity stress on plant growth. Plants under optimal growth are compared with those under salinity stress, with emphasis on changes related to osmotic and ionic stress, oxidative stress, ROS accumulation, and secondary consequences of salinity-induced stress.

Salinity poses multifaceted challenges to plants, primarily through osmotic stress, ionic toxicity, and oxidative stress. The changes are represented in Figure 1. Each of these manifests at molecular, biochemical, and structural levels, requiring coordinated mitigation by the cell’s primary protectors.

### 3.1. Osmotic and ionic stress

When plants encounter saline environments, the initial physiological disruption stems from a sharp decline in the external water potential due to elevated NaCl concentrations in the rhizosphere. This reduction in soil water restricts the plant’s ability to take up water, generating osmotic stress. The ensuing decline in turgor pressure leads to cellular dehydration, loss of cell expansion, and rapid stomatal closure, a defensive mechanism that reduces transpirational water loss but then limits CO₂ assimilation and photosynthetic efficiency (Figure 1).^11^ ^,^ ^55^

Within hours of salt exposure, plant cells experience cellular dehydration, reduced turgor pressure, and inhibition of cell expansion, particularly in the root elongation zone, where active growth is highly sensitive to osmotic imbalance (Figure 1). At the molecular level, osmotic stress activates early calcium signaling and ROS bursts in root epidermal cells. These transient signals trigger phosphorylation cascades via mitogen-activated protein kinases (MAPKs) and SnRK2s, thereby upregulating ABA-dependent stress-responsive genes and modulating aquaporin expression, thereby altering hydraulic conductivity.^56^ ^,^ ^57^ The SOS (Salt Overly Sensitive) pathway, initiated by Ca²⁺-bound SOS3 interacting with SOS2 kinase, is central to maintaining ion homeostasis by activating the plasma-membrane Na⁺/H⁺ antiporter SOS1, which extrudes excess Na⁺ from the cytoplasm.^58‐61^

With prolonged exposure, osmotic stress transitions into ionic stress as Na⁺ and Cl⁻ progressively accumulate in plant tissues via apoplastic bypass flow and symplastic transport mediated by non-selective cation channels (NSCCs) and high-affinity K⁺ transporters (HKT-type).^62‐64^ Excessive Na^+^ and Cl^-^ ions also interfere with the uptake and translocation of essential mineral nutrients such as K^+^, Ca^2+^, Mg^2+^, and NO_3_^-^ through ionic competition and disruption of membrane transport systems. This nutrient imbalance directly affects enzyme activity, membrane stability, and metabolic processes and therefore represents a primary consequence of ionic stress under salinity conditions.^65^ (Figure 1). Simultaneously, excess Na⁺ destabilizes membrane potential and disrupts metabolite transport across key intracellular organelles, including chloroplasts, mitochondria, endoplasmic reticulum, Golgi apparatus, nucleus, and peroxisomes, thereby affecting multiple metabolic pathways.^66^ ^,^ ^67^

To counteract ionic toxicity, plants activate ion compartmentalization strategies in which Na⁺ is sequestered into the vacuole via tonoplast Na⁺/H⁺ exchangers (NHX1/NHX2) driven by vacuolar H⁺-ATPases and H⁺-pyrophosphatases.^20^ ^,^ ^68^ This sequestration not only protects the cytosol but also contributes to osmotic adjustment by increasing vacuolar solute concentration, thereby maintaining cell turgor. Concurrently, compatible solutes such as proline, glycine betaine, and soluble sugars accumulate in the cytoplasm to balance osmotic pressure without interfering with enzymatic functions.^69‐72^

Over time, persistent ionic stress induces secondary oxidative damage, largely due to ROS generation from disrupted chloroplasts and peroxisomes (Figure 1). This oxidative stress interacts synergistically with osmotic and ionic components, exacerbating lipid peroxidation, protein oxidation, and DNA damage.^18^ ^,^ ^73^ Particularly in young meristematic tissues, where vacuolar capacity and antioxidant systems are not fully developed, ion toxicity can impair cell division and accelerate premature senescence.^74^ Collectively, osmotic and ionic stresses represent the primary constraints on plant productivity under salinity, as summarized in Figure 1.

### 3.2. Oxidative stress and ROS overaccumulation

Salinity stress is closely associated with oxidative stress due to excessive accumulation of ROS in different subcellular compartments. Major ROS, including superoxide anion (O₂•⁻), H₂O₂, and •OH, are generated as by-products of aerobic metabolism (Figure 1). Under saline conditions, disruption of cellular redox homeostasis enhances ROS production in chloroplasts through over-reduction of photosystem I (PSI) and photorespiration, in mitochondria through electron leakage from complexes I and III, and in peroxisomes via glycolate oxidase activity during photorespiration.^5,6,50,75‐80^

At moderate levels, ROS function as signaling molecules that regulate stress perception and activate defense pathways through redox-sensitive transcription factors. ROS-mediated signaling modulates pathways such as SOS signaling, MAPK cascades, and hormonal crosstalk involving ABA, ethylene, and JA. However, prolonged or severe salinity causes ROS accumulation beyond the detoxification capacity of the cell, resulting in oxidative damage to lipids, proteins, and nucleic acids (Figure 1). Lipid peroxidation generates malondialdehyde (MDA), disrupting membrane integrity and increasing electrolyte leakage, while protein oxidation, enzyme inactivation, and DNA damage contribute to cellular dysfunction and programmed cell death.

To maintain ROS homeostasis, plants employ both enzymatic and non-enzymatic antioxidant systems. Enzymatic antioxidants include SOD, CAT, and APX, along with glutathione reductase (GR) and the ascorbate-glutathione (AsA-GSH) cycle, which collectively regulate cellular redox balance. Non-enzymatic antioxidants such as ascorbate, glutathione (GSH), *α*-tocopherol, carotenoids, flavonoids, and phenolic compounds act as ROS scavengers and redox cofactors. Transcriptomic studies further show salinity-induced upregulation of genes encoding antioxidant enzymes, including CAT1, APX2, and FeSOD1, as well as pathways involved in phenolic antioxidant biosynthesis.^81‐83^

Salt stress also promotes peroxisome proliferation and elongation, enhancing detoxification capacity through upregulation of the PEX11 gene.^84^ ^,^ ^85^ In addition, ROS-hormone crosstalk constitutes an important regulatory mechanism during salinity stress. Elevated ROS levels stimulate ABA biosynthesis and ethylene signaling, contributing to stomatal closure, osmotic adjustment, and antioxidant defense. Salicylic acid (SA) and JA further regulate redox-responsive transcription factors such as WRKY, NAC, and DREB, thereby maintaining ROS homeostasis and facilitating stress adaptation^86‐88^ (Figure 1).

### 3.3. Secondary consequences of salinity-induced stresses

The combined effects of osmotic, ionic, and oxidative stresses lead to secondary physiological and metabolic impairments that ultimately reduce plant growth and productivity (Figure 1). One of the earliest consequences is photosynthetic inhibition caused by chloroplast damage, degradation of photosynthetic pigments, and photoinhibition of PSII and PSI.^89^ ^,^ ^90^ Excess Na^+^ in the chloroplast stroma disrupts the thylakoid proton gradient, thereby limiting ATP synthesis and carbon fixation, while stomatal closure restricts CO_2_ uptake, increasing excess excitation energy and ROS production (Figure 1).

Salinity also reprograms gene expression through activation of stress-responsive transcription factors, including DREB, MYB, bZIP, WRKY, and NAC, which regulate genes associated with osmolyte biosynthesis, ion transport, and ROS detoxification.^91‐95^ In parallel, epigenetic mechanisms such as histone acetylation and DNA methylation contribute to stress memory and adaptive responses.^96^ ^,^ ^97^ When these protective mechanisms fail to restore cellular homeostasis, prolonged oxidative and ionic stress results in membrane damage, cell death, leaf chlorosis, tissue necrosis, growth inhibition, and ultimately significant yield losses (Figure 1).

The osmotic, ionic, and oxidative disturbances described above collectively trigger extensive structural and functional adaptations in the primary cellular protectors. These adaptive modifications enable plants to maintain ionic balance, osmotic homeostasis, membrane stability, and redox regulation under saline environments. The organelle-specific responses and coordinated adaptive mechanisms are discussed in the following section.

## 4. Structural and functional changes under salinity stress

**Figure 2.**
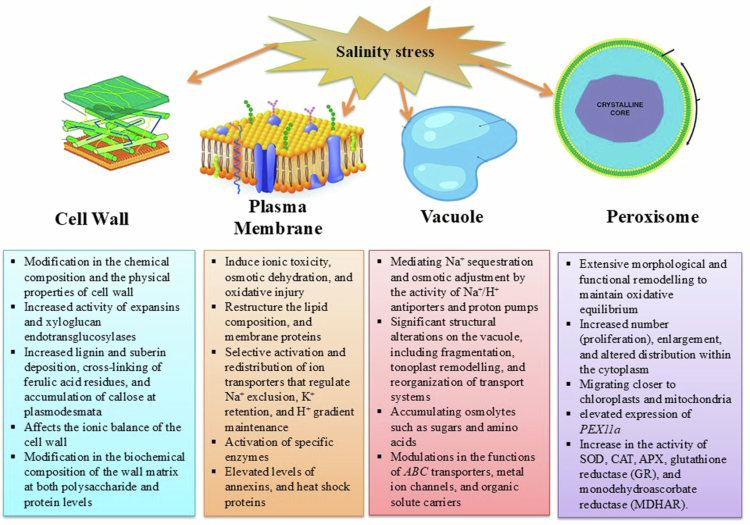
Structural modifications and associated functional changes in primary protectors under salinity stress in plants.

This section specifically focuses on the structural, biochemical, and molecular adaptations induced by salinity stress. These stress-responsive modifications enable the cell wall, plasma membrane, vacuole, and peroxisome to function as coordinated defense systems that preserve cellular integrity and metabolic homeostasis under saline conditions (Figure 2).

### 4.1. Cell wall

Under saline conditions, the cell wall undergoes extensive structural and biochemical remodeling to maintain cellular integrity, regulate ion transport, and support stress adaptation.^98^ These changes involve coordinated reorganization of cellulose, hemicellulose, pectin, and structural proteins, along with activation of wall-associated kinases (WAKs), enabling the wall to balance rigidity with osmotic flexibility.^23^ ^,^ ^32^ Salt stress alters wall composition, porosity, and mechanical properties through processes such as pectin methyl esterification and enhanced polymer cross-linking, which reduce Na^+^ influx and improve structural stability.

Salinity-induced osmotic imbalance causes loss of turgor and deformation of the wall. Under moderate stress, increased activity of expansins and xyloglucan endotransglucosylase/hydrolases (XTHs) promotes controlled wall loosening to facilitate osmotic adjustment.^99^ In contrast, prolonged or severe salinity enhances wall rigidification through lignin and suberin deposition, ferulic acid cross-linking, and callose accumulation at plasmodesmata.^24^ These modifications reduce water loss and ion leakage while stabilizing cells against osmotic shrinkage.

Salt stress also disrupts ionic balance within the cell wall. Excess Na^+^ in the apoplast competes with Ca^2+^ for binding to negatively charged pectins and uronic acids, weakening Ca^2+^-pectate cross-links and increasing wall plasticity and porosity, particularly in salt-sensitive species.^100‐102^ In salt-tolerant species, enhanced pectin methylation reduces the availability of free carboxyl groups for Na^+^ binding, thereby limiting sodium accumulation in the wall.^103^ Furthermore, boron- and calcium-mediated cross-linking of rhamnogalacturonan II (RG-II) strengthens the pectin network and helps maintain wall integrity and ion selectivity under saline conditions.^104^

These ionic adjustments transform the cell wall into an ion buffer zone, partially filtering excess Na⁺ before it reaches the plasma membrane. WAKs and receptor-like kinases (RLKs) are proposed to participate in sensing alterations in wall integrity and ionic conditions under salinity stress, thereby linking extracellular mechanical and ionic perturbations with intracellular signaling pathways.^105^ ^,^ ^106^ Under salt stress, WAKs bind to demethylated pectins and initiate Ca²⁺-dependent signaling cascades that upregulate stress-responsive transcription factors.^24^ This cell wall integrity (CWI) signaling is a critical adaptive mechanism that enables cells to detect ionic perturbations and activate downstream protective responses. Recent evidence has challenged the exclusive role of WAKs in oligogalacturonide-mediated signaling, suggesting that their functions may be context-dependent.^107^ In contrast, FERONIA (FER) receptor kinase has emerged as a key pectin sensor involved in cell wall integrity signaling and salinity adaptation.^108^ ^,^ ^109^ These findings indicate that multiple receptor systems may cooperate in perceiving wall-derived stress under saline conditions.

Salt stress reshapes the biochemical composition of the wall matrix at both polysaccharide and protein levels. In salt-tolerant cultivars, cellulose deposition is often enhanced, reinforcing wall strength and reducing permeability. Conversely, salt-sensitive plants exhibit reduced cellulose crystallinity and disorganized microfibril orientation, compromising structural integrity. Pectic polysaccharides are particularly dynamic. Increased levels of homogalacturonans (HGs) and RG-I and RG-II enhance the cell wall's hydration capacity, facilitating osmotic balance.^110‐112^ The degree of methylesterification (DM) of pectins, regulated by pectin methylesterases (PMEs) and their inhibitors (PMEIs), determines wall flexibility; low DM increases cross-linking and rigidity, whereas high DM favors elasticity.^15^ ^,^ ^113^ ^,^ ^114^

Proteomic studies have also identified increased accumulation of hydroxyproline-rich glycoproteins (HRGPs), arabinogalactan proteins (AGPs), and extensins, which form a protein–carbohydrate network that stabilizes the wall under ionic pressure.^115‐117^ These proteins not only strengthen the matrix but also participate in stress-associated signaling processes through interactions with plasma membrane-localized receptor kinases and mechanosensitive components. Growing evidence suggests that cell wall integrity signaling contributes to salinity adaptation by linking changes in wall composition and mechanics to downstream ion homeostasis and stress-responsive pathways. Thus, the plant cell wall acts as a communication hub, transmitting mechanical and ionic cues to downstream organelles, including the plasma membrane and vacuole, thereby orchestrating integrated responses such as ion compartmentalization and ROS detoxification.

### 4.2. Plasma membrane

Under salinity stress, the plasma membrane experiences severe ionic, osmotic, and oxidative damage that threatens cellular homeostasis.^14^ ^,^ ^18^ In response, plants remodel membrane lipids, transport proteins, and signaling networks to preserve membrane integrity, regulate ion selectivity, and maintain osmotic balance. Salt stress increases the proportion of unsaturated fatty acids and sterols in the membrane, thereby modulating membrane fluidity, stability, and permeability.^118^ ^,^ ^119^ These structural adjustments are coordinated through signaling pathways involving Ca^2+^, ROS, and stress hormones such as ABA, ethylene, and JA, allowing the plasma membrane to function as both a protective barrier and a stress-sensing interface.

A major adaptive response is enhanced lipid unsaturation mediated by fatty acid desaturases (FADs), particularly FAD2, FAD3, and FAD8, which increase linoleic and linolenic acid content under saline conditions.^120^ ^,^ ^121^ Increased unsaturation maintains membrane fluidity, supports protein mobility, and reduces electrolyte leakage, whereas desaturase-deficient mutants exhibit membrane rigidification, excessive ROS accumulation, and impaired Na^+^ exclusion. Salinity also promotes accumulation of membrane sterols, including sitosterol, stigmasterol, and campesterol, which form sterol-rich microdomains that stabilize the membrane and facilitate localization of signaling proteins and transporters.^17^ ^,^ ^122^ ^,^ ^123^ Increased sterol content reduces membrane permeability and enhances clustering of H^+^-ATPases, SOS1 Na^+^/H^+^ antiporters, aquaporins, and other transport proteins involved in ion and water regulation.^61^ ^,^ ^124^

Maintenance of ion homeostasis is a central plasma membrane function during salt stress. Activation of the SOS signaling pathway enables Na^+^ extrusion through SOS1 following Ca^2+^-dependent activation of SOS2-SOS3 complexes.^61^ ^,^ ^125^ Concurrently, HKT transporters, H^+^-ATPases, and H^+^-pyrophosphatases regulate Na^+^/H^+^ balance, electrochemical gradients, and cytosolic pH, while aquaporins modulate water permeability to minimize ion leakage and dehydration.^126‐128^ Together, these transport systems enable selective ion flux and osmotic adjustment under saline conditions.

The plasma membrane also serves as a signaling hub during salinity stress. NADPH oxidases generate ROS signals that activate stress-responsive pathways, while WAKs and receptor-like kinases perceive alterations in cell wall- membrane interactions and trigger Ca^2+^ signaling and MAPK cascades.^129^ ^,^ ^130^ In addition, annexins participate in membrane repair and ROS detoxification, whereas heat shock proteins such as HSP70 and HSP90 stabilize membrane-associated proteins under ionic and oxidative stress.^131‐133^

Thus, salt tolerance depends on maintaining an optimal balance between membrane fluidity and stability. Salt-tolerant plants exhibit efficient lipid remodeling and sterol adjustment that preserve selective permeability and membrane potential, whereas salt-sensitive genotypes show excessive rigidification, lipid peroxidation, and ion leakage.^17^ ^,^ ^134^ These coordinated adaptations enable the plasma membrane to integrate environmental signals, regulate ionic and osmotic balance, and initiate downstream stress responses essential for salinity tolerance.

### 4.3. Vacuole

The vacuole is a key organelle involved in ion storage, osmotic adjustment, detoxification, and maintenance of cell turgor under salinity stress. In saline conditions, it serves as a major compartment for Na⁺ sequestration, thereby protecting the cytosol from ionic toxicity while maintaining cellular water balance and metabolic stability. Salinity induces substantial structural and functional changes in the vacuole, including fragmentation, tonoplast remodeling, and reorganization of transport systems, all of which enhance vacuolar ion compartmentalization and intracellular communication.^20^

One of the earliest responses to salt stress, particularly in root meristematic and elongation zones, is vacuolar fragmentation into smaller vesicles.^135^ ^,^ ^136^ This process represents an adaptive response that improves localized ion sequestration and vacuolar trafficking under intense ionic and metabolic activity. Salt-induced fragmentation is associated with increased expression of SNARE proteins (VTI11, VAM3) and Rab-GTPases that regulate vesicle fusion and tonoplast turnover.^137^ In addition, tonoplast invaginations and multivesicular body (MVB) formation indicate enhanced endomembrane trafficking toward the vacuole.^138^

The primary defensive role of the vacuole under salinity is sequestration of Na⁺ ions away from the cytosol, thus preventing enzyme inhibition and maintaining cytosolic K⁺/Na⁺ balance.^20^ ^,^ ^62^ ^,^ ^139^ This is achieved through the coordinated activity of Na⁺/H⁺ antiporters (NHXs) and tonoplast proton pumps, which establish electrochemical gradients. Two major proton pumps energize vacuolar transport: viz., the vacuolar H⁺-ATPase (V-ATPase), which hydrolyzes ATP to pump H⁺ into the vacuolar lumen, and the vacuolar H⁺-pyrophosphatase (V-PPase), which uses pyrophosphate (PPi) as an energy source. These pumps generate a proton motive force (PMF) that drives the secondary active transport of Na⁺ and other cations via Na⁺/H⁺ exchangers. Under salt stress, transcriptional and post-translational activation of vacuolar H^+^-ATPase subunits VHA-a3 and VHA-B1, together with the vacuolar H^+^-pyrophosphatase AVP1 subunits, has been documented, correlating with increased vacuolar acidification and Na⁺ sequestration efficiency.^140^ Overexpression of *AVP1* in Arabidopsis and *OVP1* in rice enhances salt tolerance by boosting vacuolar proton gradients, increasing Na⁺ uptake into vacuoles, and improving shoot biomass under saline conditions.^141^ ^,^ ^142^ Conversely, mutants defective in V-ATPase subunits exhibit cytosolic Na⁺ accumulation and hypersensitivity to salinity.

The NHX family of tonoplast transporters plays a central role in vacuolar Na⁺ sequestration. NHX1, NHX2, and NHX4 mediate the antiport of cytosolic Na⁺ (or K⁺) in exchange for H⁺ generated by proton pumps. These exchangers maintain vacuolar ionic balance, pH homeostasis, and osmotic adjustment.^68^ ^,^ ^143^ ^,^ ^144^ In Arabidopsis*, nhx1* knockouts, Na⁺ accumulation in the cytoplasm causes oxidative injury and leaf chlorosis, demonstrating the indispensable role of vacuolar Na⁺ sequestration. In addition to NHXs, Cl⁻ channels (CLCs) and cation/H⁺ antiporters (CHXs) also contribute to vacuolar ion balance, aiding in the storage of Cl⁻ and compatible solutes.^145^ ^,^ ^146^ The integration of these systems ensures that osmotic and ionic pressures are equilibrated across the tonoplast.

Beyond Na⁺ transport, vacuoles contain a diverse set of ATP-binding cassette (ABC) transporters, metal ion channels, and organic solute carriers, which are modulated under salt exposure. The ABC subfamily C transporters mediate vacuolar sequestration of glutathione-conjugated toxins and oxidized metabolites, providing secondary detoxification.^147^ Regulatory proteins such as SOS2 kinase and 14-3-3 adapters directly interact with NHX and V-ATPase complexes, modulating their phosphorylation status and turnover.^148^ This crosstalk integrates cytosolic signaling networks with vacuolar transport function, ensuring rapid adjustment to salinity-induced ionic perturbations.

The combined activity of tonoplast pumps and exchangers allows the vacuole to serve as a controlled ionic buffer. By sequestering excess Na⁺, maintaining internal K⁺ reserves, and accumulating osmolytes such as sugars and amino acids, vacuoles help sustain turgor pressure and metabolic activity even under severe salinity. In meristematic zones, vacuolar fragmentation increases the number of tonoplasts available for Na⁺ sequestration, reducing cytosolic toxicity and enabling continued root growth. Thus, vacuolar remodeling represents a multi-level adaptation, integrating structural reorganization, transporter regulation, and energetic coupling that support cellular ion homeostasis and osmotic resilience in plants exposed to salinity.

### 4.4. Peroxisome

Peroxisomes are highly dynamic, single-membrane-bound organelles that play a pivotal role in redox metabolism, photorespiration, and lipid *β*-oxidation. As both sources and scavengers of ROS, they are key regulators of cellular oxidative balance under salinity stress. Exposure to high salinity induces extensive morphological and functional remodeling of peroxisomes, including proliferation, enlargement, and redistribution within the cytoplasm.^84^ Increased peroxisome abundance enhances ROS-detoxification capacity and fatty acid oxidation, and is associated with upregulation of peroxisomal biogenesis proteins such as PEX11A, which regulates elongation and fission, along with PEX14 and PEX5 involved in matrix protein import.^45^ ^,^ ^149^ Salinity also promotes a closer association between peroxisomes and chloroplasts and mitochondria, facilitating ROS exchange, photorespiratory metabolite transfer, and redox signaling among organelles.^5^ ^,^ ^7^

Peroxisomes contain a broad antioxidant machinery, including SOD, CAT, APX, GR, and monodehydroascorbate reductase (MDHAR), which undergo coordinated regulation during salt stress. SOD converts superoxide radicals to H₂O₂, while CAT, localized predominantly in peroxisomes, efficiently decomposes H₂O₂ into water and oxygen. Under salinity stress, CAT1 and CAT2 expression increase markedly in salt-tolerant plants, whereas delayed CAT induction in sensitive species is often associated with membrane damage and cell death.^150‐152^ APX further detoxifies residual H₂O₂ through the AsA-GSH cycle, which maintains favorable AsA/DHA and reduced glutathione (GSH)/oxidized glutathione (GSSG) ratios for cellular redox homeostasis.^7,153‐157^ The oxidized glutathione generated can subsequently be recycled in the cytosol, linking peroxisomal and cellular redox signaling.

Beyond ROS detoxification, peroxisomes contribute to metabolic adaptation under salinity through *β*-oxidation and photorespiration.^22^ Although *β*-oxidation generates additional H₂O₂, peroxisomes maintain redox balance by modulating NAD⁺/NADH ratios and increasing malate dehydrogenase (MDH) activity to facilitate redox shuttling with mitochondria.^78^ ^,^ ^80^ Collectively, peroxisomal proliferation, antioxidant reprogramming, and metabolic adjustments help in sustaining redox homeostasis, energy metabolism, and stress signaling, highlighting the peroxisome as a critical component of salinity tolerance.

## 5. Integrated efforts of the cell wall, plasma membrane, vacuole, and peroxisome in salinity tolerance

**Figure 3.**
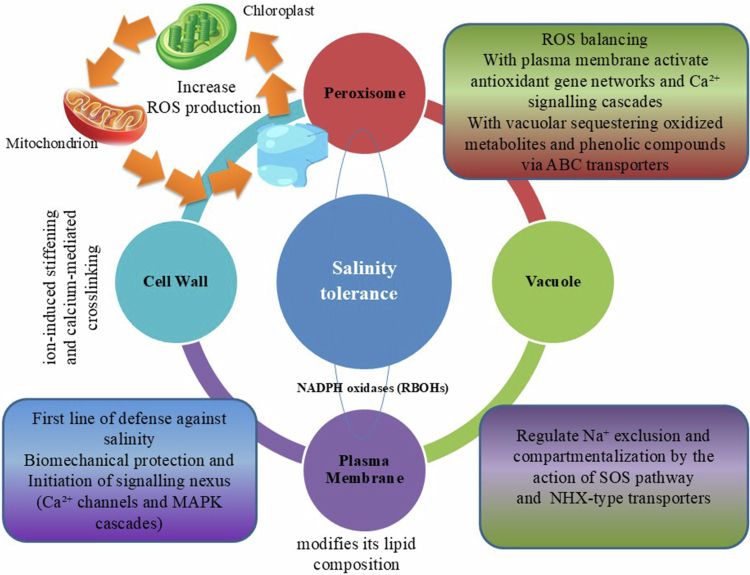
Functional integration of the primary protectors of salt stress in plants. The cell wall, plasma membrane, vacuole, and peroxisome are shown here working together as primary protectors, integrating their functions to enhance salinity tolerance.

Plant adaptation to salinity is not governed by isolated organelles but by a coordinated network of primary cellular protectors. The cell wall, plasma membrane, vacuole, and peroxisome collectively operate as an integrated structural–functional continuum, maintaining cellular homeostasis under ionic, osmotic, and oxidative stress (Figure 3). This integration reflects system-level resilience, in which biomechanical protection, ion transport regulation, and ROS detoxification are tightly interconnected to sustain plant growth under saline conditions.^6^ ^,^ ^158^

The cell wall and plasma membrane together constitute the first line of defense (Figure 3). The cell wall undergoes ion-induced stiffening and calcium-mediated crosslinking, which enhances mechanical strength and limits ion penetration.^15^ ^,^ ^16^ Simultaneously, the plasma membrane modifies its lipid composition to preserve fluidity and selective permeability under stress conditions.^18^ This interface also serves as a signaling nexus, where mechanosensitive channels and receptor-like kinases trigger Ca²⁺ influx and MAPK cascades, thereby initiating downstream stress responses.^17^ ^,^ ^104^ These signaling pathways regulate transcriptional networks that control ion transporters and antioxidant defenses, thereby linking structural integrity to biochemical adaptation.^6^

The coordinated interaction between the plasma membrane and vacuole is central to maintaining ionic homeostasis (Figure 3). Excess Na^+^ is extruded from the cytosol through plasma membrane transport systems, such as the SOS pathway, while vacuolar sequestration, mediated by NH-type antiporters and energized by V-ATPase and V-PPase proton pumps, maintains low cytosolic Na^+^ concentrations and osmotic balance.^19^ ^,^ ^20^ Additionally, the cell wall contributes to ionic buffering by adsorbing Na^+^ and Cl^-^ ions via negatively charged polysaccharides, thereby stabilizing the apoplastic environment and reducing ionic stress at the membrane interface.^14^

Salinity stress also leads to enhanced ROS generation in chloroplasts and mitochondria (Figure 3), which can disrupt cellular redox balance and damage macromolecules.^21^ ^,^ ^22^ The plasma membrane contributes to ROS signaling via NADPH oxidases (RBOHs), which generate controlled bursts of ROS that act as secondary messengers to activate Ca^2+^ signaling and stress-responsive gene expression.^6^ These ROS signals are subsequently detoxified in peroxisomes, which act as central hubs of ROS metabolism through enzymes such as CAT, SOD, and APX. In parallel, the vacuole plays a supportive role in redox regulation by sequestering oxidized metabolites and phenolic compounds via ABC subfamily C transporters, thereby minimizing oxidative damage in the cytosol.

Thus, through spatial compartmentalization and signal transduction, the peroxisome–vacuole–membrane axis ensures controlled ROS homeostasis while minimizing cellular damage. Communication among the cell wall, plasma membrane, vacuole, and peroxisome is mediated by ion fluxes, redox signals, and vesicular trafficking. Evidence from proteomics and live-cell imaging suggests that tethering complexes and contact sites facilitate metabolite and signal exchange.

## 6. Interactions of primary protectors with other organelles in salinity stress tolerance

**Figure 4.**
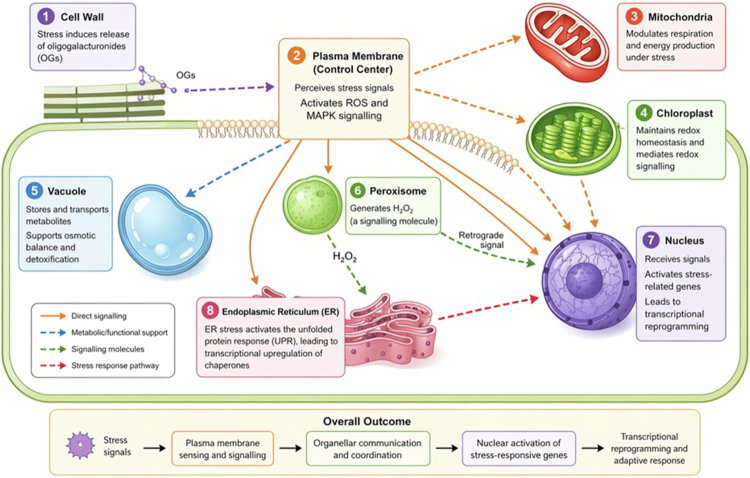
Cross-talk between primary protectors and other cellular structures in salinity stress tolerance.

Salinity tolerance is a whole-cell phenomenon in which the integrated activities of structural barriers and metabolic organelles ensure survival under osmotic, ionic, and oxidative constraints. While the cell wall, plasma membrane, vacuole, and peroxisome serve as the primary protectors, their effectiveness depends on dynamic crosstalk with chloroplasts, mitochondria, the endoplasmic reticulum (ER), and the nucleus (Figure 4).

These interactions sustain energy flow, maintain ionic balance, modulate redox signaling, and coordinate gene expression, establishing a multi-layered defense network against salt-induced cellular dysfunction. Cell wall-derived fragments, such as oligogalacturonides (OGs), act as damage-associated molecular patterns (DAMPs) that are perceived by plasma membrane receptors, triggering systemic ROS and MAPK signaling.^159^ ^,^ ^160^ This mechanical and chemical information from the cell wall is transmitted inward, influencing mitochondrial respiration, chloroplast redox poise, and nuclear transcriptional reprogramming of stress-related genes. Through these pathways, the cell wall not only acts as a passive ion buffer but also as a primary communicator between the extracellular environment and intracellular organelles, linking structural stress perception to metabolic and transcriptional regulation.

The plasma membrane serves as a central hub coordinating ion transport, signal transduction, and ROS communication between organelles. Under salt stress, membrane depolarization triggers cytosolic Ca²⁺ transients, which are decoded by calcineurin B-like proteins (CBLs) and CBL-interacting kinases (CIPKs).^161^ ^,^ ^162^ These kinases regulate SOS1, NHX, and HKT transporters, facilitating Na⁺ extrusion and K⁺ retention. The plasma membrane also interacts with the ER via ER-PM contact sites, where lipid and calcium exchange occur. These contacts help stabilize membrane fluidity under salt stress, ensuring proper trafficking of ion channels, aquaporins, and H⁺-ATPases, which are synthesized and folded in the ER. Additionally, metabolite shuttling occurs between vacuoles and other organelles via tonoplast transporters and vesicular trafficking. This includes the export of sugars and amino acids required for osmotic adjustment and antioxidant synthesis in the cytoplasm and plastids. Through such cooperation, the vacuole ensures a homeostatic triad involving osmotic stabilization, metabolic buffering, and support for detoxification in chloroplasts and mitochondria.

The ER and the nucleus serve as regulatory centers that integrate stress perception and response. The ER ensures the proper folding of membrane and transporter proteins, including aquaporins, H⁺-ATPases, and ion channels, which are essential for vacuolar and plasma membrane function. Under salinity, ER stress activates the unfolded protein response (UPR), leading to the transcriptional upregulation of chaperones (BiP, Calnexin) and protein disulfide isomerases.^163‐165^ Meanwhile, the nucleus acts as the command center, coordinating transcriptional reprogramming in response to ionic and oxidative signals transmitted from the cell wall and membrane.^38^ ^,^ ^166^

Transcription factors such as DREB2A, NAC, bZIP, and WRKY are activated by MAPK cascades and Ca²⁺-dependent kinases.^167‐169^ These TFs regulate genes involved in osmolyte biosynthesis, antioxidant production, transporters (SOS1, NHX1, and HKT1), and cell wall-modifying enzymes. Peroxisome-derived H₂O₂ serves as a retrograde signal to the nucleus, modulating gene expression via ZAT and AP2/ERF transcription factors. This ensures redox homeostasis is transcriptionally reinforced during sustained salinity exposure. Additionally, the ER and peroxisomes physically interact through membrane contact sites (MCSs), which coordinate lipid exchange and peroxisome biogenesis, thereby maintaining the integrity of peroxisomal membranes under oxidative stress.^170‐172^ These structural and regulatory communications ensure that stress responses remain coherent and energy-efficient across the cell.

An integrated view reveals that plant cell organelles under salinity form a dynamic communication network. The cell wall detects and relays mechanical and ionic perturbations. The plasma membrane interprets external signals into electrochemical and ROS-based cascades. The vacuole buffers ion and osmotic imbalances through sequestration and storage. The peroxisome modulates ROS detoxification and redox signaling. Chloroplasts and mitochondria generate energy and ROS signals to support adaptive metabolism. The ER and the nucleus regulate protein turnover and transcriptional reprogramming. This orchestration ensures that each cellular structure contributes specialized yet interdependent functions, thereby maintaining cellular homeostasis under fluctuating salinity conditions. In modern systems-biology terms, salinity tolerance emerges from the networked resilience of the plant cell rather than from isolated defensive modules.

## 7. Future directions

Salinity stress imposes severe physiological, biochemical, and structural challenges on plant cells by disrupting water balance, ion homeostasis, and redox stability. In response, the cell wall, plasma membrane, vacuole, and peroxisome function as interconnected primary protectors that collectively maintain cellular integrity and metabolic balance. These organelles coordinate ion regulation, ROS detoxification, osmotic adjustment, and stress signaling through continuous interaction with the nucleus, ER, chloroplasts, and mitochondria, highlighting the integrated nature of cellular stress adaptation.

This coordinated defense system integrates ion homeostasis, osmotic adjustment, ROS detoxification, and stress signaling. The cell wall and plasma membrane regulate Na^+^ exclusion and selective ion transport through components such as SOS1 and HKT1, while vacuoles sequester Na^+^ via NHX antiporters and maintain osmotic balance through compatible solute accumulation. Concurrently, aquaporins and cell wall remodeling support cellular hydration. Peroxisomes, together with chloroplasts and mitochondria, maintain ROS homeostasis through antioxidant systems involving CAT, SOD, and APX, while NADPH oxidases and cell wall peroxidases mediate ROS signaling. These responses are coordinated through Ca^2+^ signaling, MAPK cascades, and transcription factors such as WRKY, DREB, and NAC, with WAKs and RLKs linking stress perception to downstream adaptive responses.

Despite substantial progress, important gaps remain in understanding how these organelles function collectively under salinity stress. Future research should focus on membrane contact sites linking the plasma membrane, ER, vacuole, and peroxisomes, as well as on the mechanisms by which CWI sensors transmit stress signals to intracellular compartments. Advanced imaging, proteomics, phospho-signaling studies, and integrated omics approaches combining transcriptomics, metabolomics, and ionomics will improve understanding of organelle coordination during stress adaptation. Targeted manipulation of ion transporters, antioxidant enzymes, and cell wall remodeling proteins, together with synthetic biology approaches, may further enhance salinity tolerance. Comparative studies between halophytes and glycophytes could also identify structural and molecular traits associated with natural salt tolerance, providing valuable insights for crop improvement and regenerative agricultural strategies.
